# 518. Wound Healing Outcomes with Intact versus Particularized Fish Skin Grafts

**DOI:** 10.1093/jbcr/irag033.177

**Published:** 2026-04-06

**Authors:** Maximilian Moshammer, Anna-Lisa Pignet, Ines Foessl, Manuel Prevedel, Anna Schwarz, Ana Villa-Garcia, Lars-Peter Kamolz, Elisabeth Hofmann, Petra Kotzbeck

**Affiliations:** COREMED – Center for Regenerative and Precision Medicine, JOANNEUM RESEARCH Forschungsgesellschaft mbH, Graz, Austria, Graz, Steiermark; Division of Plastic, Aesthetic and Reconstructive Surgery, Department of Surgery, Medical University of Graz, Austria, Graz, Steiermark; COREMED – Center for Regenerative and Precision Medicine, JOANNEUM RESEARCH Forschungsgesellschaft mbH, Graz, Austria, Graz, Steiermark; COREMED – Center for Regenerative and Precision Medicine, JOANNEUM RESEARCH Forschungsgesellschaft mbH, Graz, Austria, Graz, Steiermark; COREMED – Center for Regenerative and Precision Medicine, JOANNEUM RESEARCH Forschungsgesellschaft mbH, Graz, Austria, Graz, Steiermark; COREMED – Center for Regenerative and Precision Medicine, JOANNEUM RESEARCH Forschungsgesellschaft mbH, Graz, Austria, Graz, Steiermark; Division of Plastic, Aesthetic and Reconstructive Surgery, Department of Surgery, Medical University of Graz, Austria, Graz, Steiermark; COREMED – Center for Regenerative and Precision Medicine, JOANNEUM RESEARCH Forschungsgesellschaft mbH, Graz, Austria, Graz, Steiermark; COREMED – Center for Regenerative and Precision Medicine, JOANNEUM RESEARCH Forschungsgesellschaft mbH, Graz, Austria, Graz, Steiermark

## Abstract

**Introduction:**

Acellular fish skin grafts (FSG) have proven effective in wound healing, accelerating epithelialization and reducing contraction. While clinical benefits are established, the underlying cellular and molecular mechanisms remain insufficiently defined. This study investigated the effects of FSG on wound healing in a porcine full-thickness skin defect model, focusing on healing kinetics, tissue formation, and inflammatory modulation.

**Methods:**

Full-thickness excisional wounds were created in male Landrace pigs (mean weight 28.8 ± 2.8 kg, n = 9) and treated with intact sheet FSG, particularized FSG, or left untreated. Wounds were evaluated over 21 days by clinical documentation, thermography, histology and molecular analysis of cytokines and wound contraction markers (αSMA/ACTA2). Secondary dressings (foam vs. fatty gauze) were also compared.

**Results:**

FSG-treated wounds showed accelerated tissue formation with a 1.7–2.0-fold increase in thickness by day 5–9 compared to controls. Epithelialization was faster, and wound contraction was reduced in FSG groups. Cytokine profiling revealed early upregulation of IL-1β, IL-6, and IL-8 at day 5, followed by decreased IL-6 and increased IL-10 at day 9, consistent with a transition from pro-inflammatory to regenerative signaling. αSMA/ACTA2 expression kinetics indicated reduced contraction in intact FSG-treated wounds compared to controls. Secondary dressing choice partially influenced cytokine expression, with gauze favoring a more sustained pro-inflammatory response than foam.

**Conclusions:**

In a translational pig model, both intact and particularized FSG accelerated wound healing, promoted tissue formation, modulated inflammatory dynamics and reduced contraction. These findings highlight the regenerative role of FSG in complex wound management.

**Applicability of Research to Practice:**

Standardized porcine wound models provide valuable insights into wound healing mechanisms, as porcine skin closely resembles human skin in structure and physiology. Findings from such models therefore demonstrate a high degree of translatability to clinical practice, supporting the relevance of FSG as a therapeutic option in human wound care.

**Funding for the study:**

N/A.

